# Mechanically Interlocked Hydrogel–Elastomer Strain Sensor with Robust Interface and Enhanced Water—Retention Capacity

**DOI:** 10.3390/gels8100625

**Published:** 2022-09-30

**Authors:** Wenyu Zhao, Zhuofan Lin, Xiaopu Wang, Ziya Wang, Zhenglong Sun

**Affiliations:** 1 School of Science and Engineering, The Chinese University of Hong Kong, Shenzhen 518172, China; 2 Center for Stretchable Electronics and Nano Sensors, Key Laboratory of Optoelectronic Devices and Systems of Ministry of Education, College of Physics and Optoelectronic Engineering, Shenzhen University, Shenzhen 518060, China; 3 Shenzhen Institute of Artificial Intelligence and Robotics for Society, Shenzhen 518129, China

**Keywords:** hydrogel-elastomer hybrid, interlocked interface, human motion monitoring

## Abstract

Hydrogels are stretchable ion conductors that can be used as strain sensors by transmitting strain-dependent electrical signals. However, hydrogels are susceptible to dehydration in the air, leading to a loss of flexibility and functions. Here, a simple and general strategy for encapsulating hydrogel with hydrophobic elastomer is proposed to realize excellent water-retention capacity. Elastomers, such as polydimethylsiloxanes (PDMS), whose hydrophobicity and dense crosslinking network can act as a barrier against water evaporation (lost 4.6 wt.% ± 0.57 in 24 h, 28 °C, and ≈30% humidity). To achieve strong adhesion between the hydrogel and elastomer, a porous structured thermoplastic polyurethane (TPU) is used at the hydrogel-elastomer interface to interlock the hydrogel and bond the elastomer simultaneously (the maximum interfacial toughness is over 1200 J/m^2^). In addition, a PDMS encapsulated ionic hydrogel strain sensor is proposed, demonstrating an excellent water-retention ability, superior mechanical performance, highly linear sensitivity (gauge factor = 2.21, at 100% strain), and robust interface. Various human motions were monitored, proving the effectiveness and practicability of the hydrogel-elastomer hybrid.

## 1. Introduction

Hydrogels consist of crosslinked polymeric networks and retained water, allowing them to behave like solids and fluids [1,2]. The crosslinked polymeric networks of the hydrogels shape them into a stable and integrated structure, and the deformability and stretchability of the polymer molecular chain exhibit flexibility and softness at a macroscopic level [3]. On the other hand, the water contained in the hydrogels contributes liquid-like properties, such as optical transparency, dissolving capacity, permeability, etc. When ions are introduced into hydrogels, they behave like “solid aqueous electrolytes”, which are preferred for wearable strain sensing due to their suitable flexibility (typically can be readily tuned from 1 kPa to 100 kPa, close to human skin) and biocompatibility [1,4,5]. However, hydrogel-based wearable electronics usually undergo dehydration within a few hours after exposure to the ambient environment, leading to the failure of the mechanical properties and functions, thus seriously limiting their service life [6]. Therefore, improving the water-retention capacity of hydrogels is of great significance to improving their stability, prolonging their service life, and expanding their other practical applications.

Alcohols, such as glycerol (G), glycol, or sorbitol, are often used to enhance the water-retention capacity of hydrogels due to their low vapor pressure and stronger hydrogen bonding in the G-water mixture [7,8,9]. For example, Han et al. reported a hydrogel with long-lasting moisture achieved by copolymerizing acrylamide and acrylic acid monomers in a G-water binary-solvent system [10]. Its good mechanical properties, conductivity, and adhesiveness could last at least 30 days. However, it is necessary to replace water with a large amount of G to obtain substantial water retention (usually need >50 wt.% of G), which inevitably leads to changes in the physical and chemical properties of hydrogels, such as strength deterioration, toughness deterioration, ion conductivity decline, etc. [10]. 

Another solution, encapsulating hydrogels with a polymer coating to retain water, is also proposed [11,12,13,14]. The hydrophobic polymer coating provides a barrier against water evaporation, which is universal to any composition of hydrogels and does not add other substances to hydrogels. However, hydrogels (hydrophilic) and elastomers (hydrophobic) often have low adhesion energy, which can cause interface separation under tensile strain. To improve the adhesion energy of the hydrogel-elastomer interface, Yuk et al. adopted 3-(trimethoxysilyl) propyl methacrylate as the chemical binding agent and achieved a strong bond between a PAAm-alginate gel and polymer surfaces [15]. The prepared hydrogel-Ecoflex hybrid exhibited excellent anti-dehydration properties, without a noticeable change in weight over 48 h (24 °C and 50% humidity). Inspired by the structure of the skin, Zhao et al. developed an organogel-hydrogel hybrid to maintain water content by introducing double bonds onto the hydrogel surface as anchoring points to copolymerize with the organogel [16]. Similarly, Zhu et al. proposed a double-hydrophobic-coating-encapsulated hydrogel with enhanced water-retention capacity [17]. Based on the coated stearic acid hydrophobic layer, the second hydrophobic oil layer was infused into the polymer coating to build the double-layered structure. As a result, the weight retention of the double-layer-coated hydrogel was higher than 70 wt.% after air-drying for 5 days (25 °C and 30 RH%). However, the formation of this covalent anchoring depends on the surface treatment of the used elastomer and hydrogel, and the corresponding chemical binding agent, therefore, is not universally applicable.

**Figure 1:**
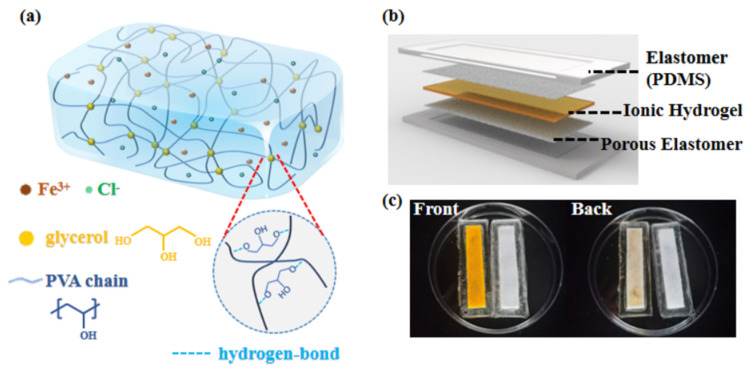
Scheme structure of (**a**) PVA-G-FeCl_3_ hydrogel and (**b**) PDMS-encapsulated ionic hydrogel. (**c**) Photos of PDMS-encapsulated PVA-G-FeCl_3_ and PVA-G hydrogel (for easy viewing, only the back side has a porous layer).

This study proposed a simple and general strategy for encapsulating hydrogels by hydrophobic porous elastomers that results in excellent binding energy between the hydrogel-elastomer interface, while also improving the water retention of the hydrogel. As shown in Figure 1a, a PVA hydrogel with G as a crosslinking agent and FeCl_3_ as an electrolyte (PVA-G-FeCl_3_) was prepared. PDMS, which possesses sufficient stretchability and adjustable modulus to fit the hydrogel, was selected as the encapsulation layer to further improve the water retention and robustness of the hydrogel. Then, the PVA-G-FeCl_3_/polydimethylsiloxane (PDMS) hybrid was developed as an ionic hydrogel strain sensor (IHSS), as demonstrated in Figure 1b, c. The thermoplastic polyurethane (TPU) porous substrate, which was prepared by the sacrificial template method, was used as the adhesive for both the PDMS and the hydrogel. Consequently, in the absence of excessive additives (G ≤ 30 wt.%) or any surface modifications of hydrogel, the sensor exhibits a good water-retention capacity (lost 4.6 wt.% ± 0.57 in 24 h, 28 °C, and ≈30% humidity), high interfacial toughness (over 1200 J/m^2^), and high sensitivity (gauge factor (GF) = 2.21). Overall, this study addresses the challenge of developing hydrogel-elastomer hybrid materials and enables novel applications in various fields by introducing a new approach that leverages the unique and complementary strengths of hydrogels and elastomers.

## 2. Results and Discussion

### 2.1. Anti-Dehydration Property of the Hydrogels

**Figure 2:**
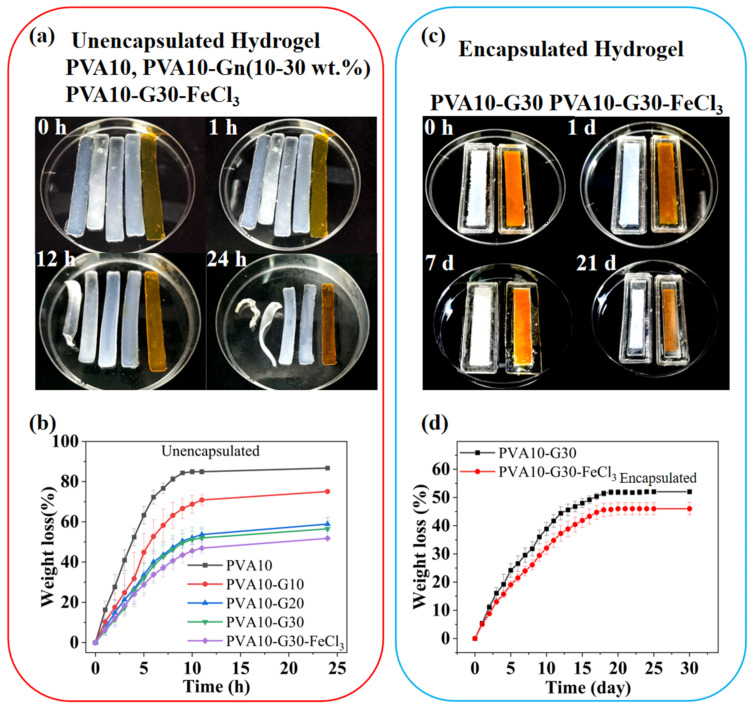
(**a**) Comparison of PVA10, PVA10-Gn, and PVA10-G30-FeCl_3_ hydrogels exposed in the air at the beginning, after an hour, after half a day, and after a day. (**b**) Weight change of the PVA10, PVA10-Gn, and PVA10-G30-FeCl_3_ hydrogels as a function of time. (**c**) Comparison of PDMS-encapsulated PVA10-G30 and PVA10-G30-FeCl_3_ hydrogels exposed in the air at the beginning, after a day, after 7 days, and after 21 days. (**d**) Weight change of the PDMS-encapsulated PVA10-G30 and PVA10-G30-FeCl_3_ hydrogels as a function of time.

Unencapsulated PVA-G hydrogels exhibit a certain water-retention capacity that is highly dependent on the G mass percentage of the mixture (G wt.%). Compared to the bare hydrogels, the hydrogel encapsulated by PDMS exhibited suitable anti-dehydration properties. Figure 2a,b show that the pure hydrogel with a PVA mass percentage of 10 wt.% (PVA10, the number represents the mass percentage of the substance, same as follows) is susceptible to dehydration in the air at room temperature (RT, 28 °C and ≈30% humidity). Its shape became unsustainable after a few hours and lost almost all its moisture after 24 h. With the use of G and its mass percentage increase, the water-retention capability of PVA10-Gn (n including 10 wt.%, 20 wt.%, and 30 wt.% of G, the same below) hydrogels is gradually improved. In addition, their ability will be further enhanced by adding hygroscopic salt such as FeCl_3_ on this basis; however, they still cannot avoid losing a large amount of water in a few hours until 24 h [18]. Compared with pure hydrogels, the PVA10-G30-FeCl_3_ hydrogel still retains some water, but in the form of hydrates. For ionic hydrogels, the corresponding decrease in water content in a short time will change their mechanical and electrical properties during use, and this is unacceptable. When hydrogels (PVA10-G30 and PVA10-G30-FeCl_3_) are coated with PDMS, as Figure 2c,d show, their water-retention ability is certified by the fact that the weight of hydrogels has minor drift (about 5% in 24 h). As the exposure time becomes longer, their moisture evaporates at a relatively slower rate and eventually reaches equilibrium after about 21 days (loses 51.88% ± 0.97 for PVA10-G30 and 46.01% ± 2.19 for PVA10-G30-FeCl_3_). 

**Figure 3:**
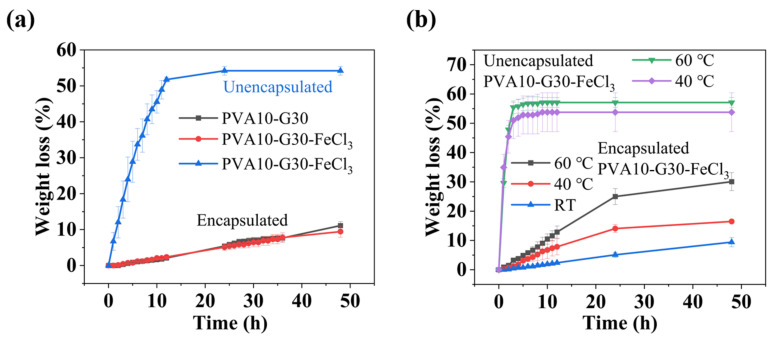
(**a**) Comparison of water retention between encapsulated and unencapsulated hydrogels exposed to air at room temperature (RT). (**b**) Comparison of water retention between encapsulated and unencapsulated hydrogels exposed to air at RT, 40 °C, and 60 °C.

For more visualization, Figure 3a compares the weight change of encapsulated and unencapsulated hydrogels (PVA10-G30 and PVA10-G30-FeCl_3_) at room temperature. The PDMS coating significantly improved the water retention of the hydrogels within 48 h; therefore, it can greatly improve the service life of hydrogel-based sensors. In addition, almost all hydrogels cannot resist hot environments and do not have long-term stability at high temperatures, thus limiting the use of hydrogels, as well. Figure 3b shows that unencapsulated hydrogels dry rapidly when exposed to high temperatures (40 °C or 60 °C) and completely lose all their water within 5 h. However, the encapsulated PVA10-G30-FeCl_3_ hydrogels exhibit better temperature resistance (loses 14% ± 1 of water in 24 h and 16% ± 1 in 48 h at 40 °C; and loses 25% ± 3 in 24 h and 30% ± 3 in 48 h at 60 °C), indicating the comprehensive water-retention capacity of PDMS coating.

### 2.2. Robustness of the Hydrogel-Elastomer Hybrid

#### 2.2.1. Interfacial Toughness of the Hydrogel-Elastomer Hybrid

**Figure 4:**
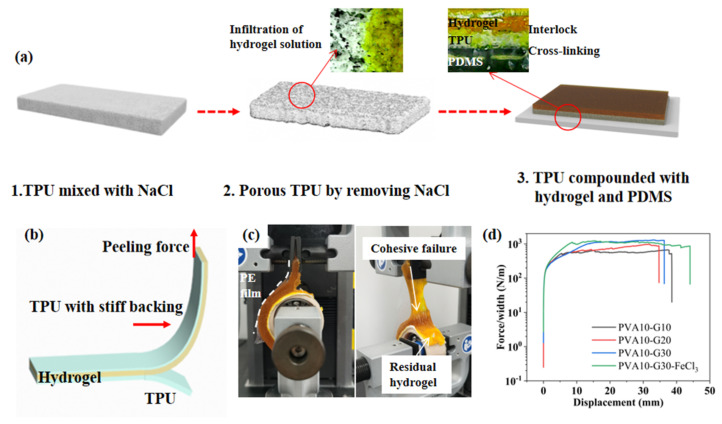
(**a**) The schematic diagram of the preparation of hydrogel-TPU-PDMS hybrid. (**b**) The schematic diagram of the 90°—peeling test. A stiff backing is introduced to prevent elongation of the hydrogel sheet along the peeling direction. (**c**) Photos of the hydrogel-TPU interface during the peeling test. The tough hydrogel undergoes a cohesive failure during the peeling test, leaving a thin residual layer of hydrogel on the TPU substrates. (**d**) The measured peeling forces per width of the hydrogel sheets for hydrogel-TPU hybrids.

The main challenge of encapsulating hydrogels with elastomers for water retention is to achieve strong adhesion between hydrogels and hydrophobic elastomers (typically, their adhesion energy is lower than 1 J/m^2^) [19], because poor interfacial adhesion may result in stability issues during long-term usage [20]. In this paper, a simple yet general method is proposed to form an extremely robust interface by using porous TPU at the interface as an adhesive for both hydrogels and PDMS. Figure 4a demonstrates the fabrication-process flow of the porous TPU and the hybrid. First of all, TPU slurry was mixed with NaCl particles uniformly and formed by a molding method. After heat treatment and removal of NaCl, the porous TPU layer was obtained. Then the porous TPU layer was placed on the uncured PDMS (the ratio of crosslinker to monomer is 1:20) and heated to 100 °C for an hour. During curing, the PDMS that partially permeated the TPU void bonded them together. Then the un-crosslinked PVA10-G30-FeCl_3_ solution was spread on the TPU layer. Under gravity, the flowing hydrogel can soak into the micropores on the surface of the TPU layers, resulting in a large interfacial area. After frozen crosslinking, the hydrogel can be mechanically interlocked at the surface of the porous TPU layers to achieve good adhesion. 

To quantify the robustness of the hydrogel-elastomer hybrid, the standard 90°—peeling test was used to measure the interfacial toughness of hydrogel sheets bonded on porous TPU substrates, as illustrated in Figure 4b, c. The bottom TPU layer was attached to a bearing by double-sided adhesive to ensure 90° during the test. The upper layer was stuck with a thin polyethylene (PE) film as the stiff backing to prevent the elongation of the hydrogel during the test. In Figure 4c, the cohesion failure occurs on the hydrogel of the hybrid rather than the hydrogel-TPU interface, leaving a hydrogel residual layer on the TPU matrix. This suggests that the fracture energy of the hydrogel-TPU interface is greater than that of the hydrogel itself. In addition, Figure 4d depicts the measured corresponding interfacial toughness that reaches over 1000 J/m^2^ for both PVA10-Gn and PVA10-G30-FeCl_3_; which is tough compared to that reported in previous reports [21,22,23]. These results suggest that the proposed method is capable of achieving consistently high interfacial toughness for hydrogels bonded onto elastomers.

#### 2.2.2. Modulus Adaptation of the Hydrogel-Elastomer Hybrid

**Figure 5:**
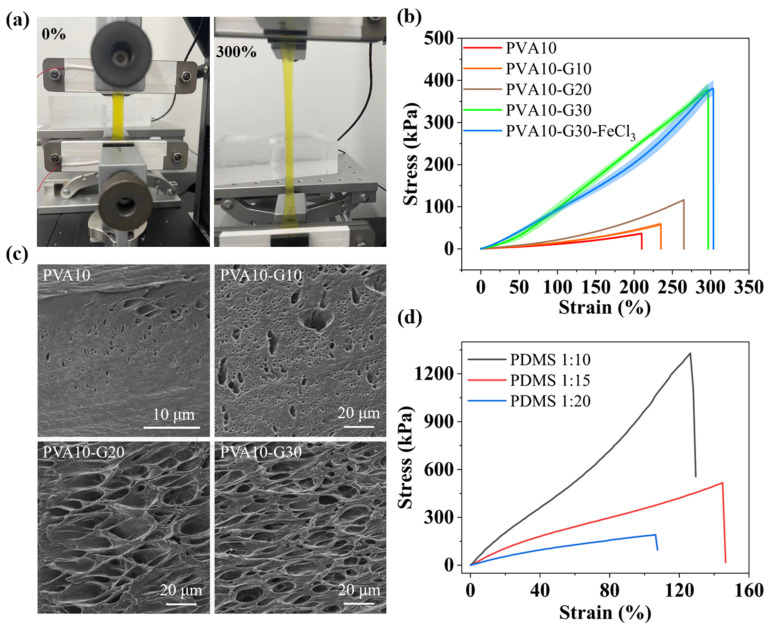
(**a**) Photos of the original PVA10-G30-FeCl_3_ hydrogel and the hydrogel after being stretched to 300% strain. (**b**) Comparison of the stress-strain curve of PVA10, PVA10-Gn, and PVA10-G30-FeCl_3_ hydrogels. (**c**) SEM image for freeze-dried PVA10 and PVA10-Gn hydrogels. (**d**) Comparison of the stress-strain curve of PDMS with the ratio of crosslinker to the monomer of 1:10, 1:15, and 1:20.

The modulus adaptation between the hydrogel and elastomer substrate is another factor affecting the stability of the IHSS. An excessive modulus difference will also lead to interface separation, thus decreasing sensor sensitivity and accuracy. The mechanical properties of PVA10-Gn hydrogels are greatly affected by the number of freeze-thaw crosslinking reactions and G content. A tensile test, as shown in Figure 5a,b, was performed to evaluate the mechanical properties of the hydrogels. The maximum tensile properties of the hydrogel reached more than 300% strain. Pure PVA10 hydrogels exhibit deficient mechanical strength (11.38 kPa), and the elongation at break was relatively low (200%). In contrast, with the introduction of G, the tensile strength, elongation at break, and toughness (area under the stress-strain curve; see Figure 5b) of PVA10-Gn hydrogels are improved significantly, and this improvement is mainly dependent on the G content. In addition, the PVA10-G30 hydrogel exhibits the highest strength (95.52 kPa) and strain (300%). This is because, in the PVA-G system, G molecules can provide three hydroxyl groups that can form multiple hydrogen bonds with the PVA chain. Under low strain, these hydrogen-bond breaks can effectively dissipate energy, while, at high tensions, PVA crystals that act as physical crosslinking points can rearrange and break to dissipate energy. However, the presence of FeCl_3_ has little effect on the mechanical properties of hydrogels.

In addition, G has a significant influence on the microstructure of the hydrogel network as well. The strong hydrogen bond interaction between G and PVA molecules can significantly reduce the chain mobility of PVA molecules, resulting in a significant reduction in crystal size, which will lead to the formation of nanofiber polymer networks. Therefore, the PVA and PVA-G hydrogels were analyzed by scanning electron microscopy (SEM) to understand the effect of G on the microstructure changes of PVA hydrogels, as shown in Figure 5c.

The freeze-dried PVA10 hydrogel sample was porous, with a few microns in diameter. When the ratio of G to water is greater than 1, the PVA organic hydrogel exhibits a hierarchical porous structure with multiple interconnected micropores (about 50 nm). With increasing mass percentages of G added, the hydrogels showed a hierarchical porous structure, with the large ones having a diameter of ~20 microns and the small ones only a few microns, and the holes were interconnected between each other. These results indicate that, in PVA-G hydrogels, more severe and abundant entanglement of PVA molecular chains occurs, resulting in the formation of nanoscale PVA networks. This microporous structure improves the mechanical properties of the hydrogel and facilitates ion conduction in the hydrogel, which is of great significance for strain sensors [24]. To match the modulus of the hydrogel, the ratio of crosslinker to monomer was adjusted for tuning the mechanical properties of PDMS. As Figure 5d depicts, when the ratio of crosslinker to monomer decreased from 1:10 to 1:20, the modulus of PDMS decreased significantly, from 964.89 kPa to 182.94 kPa.

### 2.3. Mechanism and Properties of the IHSS

**Figure 6:**
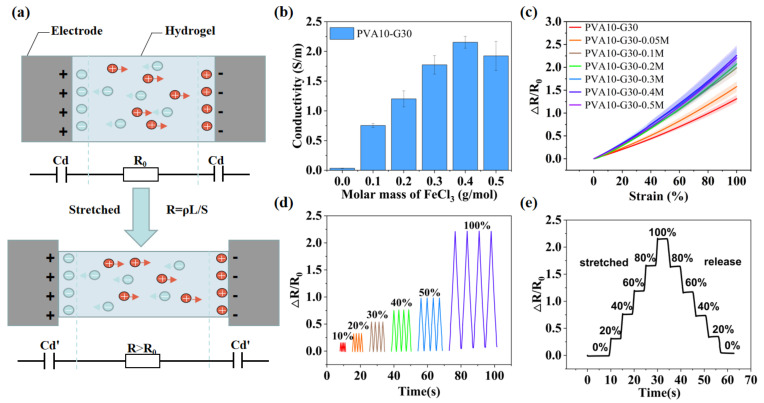
(**a**) Schematic diagram of the working principle of the IHSS. The electrical double layer (EDL) forms at the electrode and electrolyte interface. The EDL behaves like a capacitor and is considered a constant to simplify the model. (**b**) The conductivity of PVA-G-FeCl_3_ hydrogels with different ion concentrations. The critical point concentration is about 0.4 M. (**c**) Comparison of relative resistance change of PVA-G-FeCl_3_ hydrogels with ion concentration from 0 M to 0.5 M. The strain range is 0–100%. (**d**,**e**) Cyclic stability tests of IHSS under strain from 10% to 100%.

Figure 6a depicts the working mechanism of the IHSS. When energized, non-electrostatic adsorption of ions on the electrode surface occurs in the hydrogel, resulting in the formation of an electric double layer at the interface on both sides of the hydrogel electrode [3]. For simplicity, they are considered two constant capacitors during the elongation of IHSS under tension. Therefore, only changes in hydrogel bulk resistance need to be considered. When the IHSS is stretched, the resistance increases according to the definition R = ρΔL/S’, where R is the resistance of the hydrogel film, ΔL is the elongation of the stress, S’ is the cross-sectional area after being stretched, and ρ is the ionic resistivity. Since the deformation of ionic hydrogel results from the change of the polymer network and water-molecule configuration, the ionic conductivity can be regarded as constant during the deformation process and is mainly determined by the ion concentration. As shown in Figure 6b, PVA10-G30 hydrogel without ions is also somewhat conductive (0.03 S/m) due to the hydrolysis of ions in the PVA-G system. The incorporation of the electrolyte (FeCl_3_) significantly enhanced the conductivity of the PVA-G hydrogel. With the increase of the molar mass of FeCl_3_ added, the conductivity of hydrogel continued to increase until it reached a plateau at the concentration of 0.4 M (2.15 S/m). Because ions act as charge carriers in the hydrogel, the increase in electrolyte concentration means that there are more ions in the gel per unit volume, resulting in the number of charges per unit time per unit cross-sectional area going up under the electric field. However, ion transportation is limited by the configuration of the hydrogel crosslinking network. When the ion concentration rises to a critical value, the number of charges per unit time per unit cross-sectional area does not change with the increase of electrolyte concentration anymore, and a plateau appears [25,26,27,28]. The strain sensitivity of hydrogel-based sensors was calculated by the *GF*, as defined by the ratio of the relative resistance change rate to the applied strain:

(1) $$\mathrm{GF}=\frac{\Delta R/R_{0}}{\varepsilon }$$

In the whole strain range (0–100%), the resistance change rate (Δ*R*/*R*_0_) of PVA10-Gn and PVA10-G30-FeCl_3_ hydrogels increased linearly with the increase of tensile strain, and it also increased with the concentration of FeCl_3_ until it reached a plateau at the concentration of 0.4 M (*GF* = 2.21). Thus, the PVA10-G30-FeCl_3_ (0.4 M) hydrogel is used as the IHSS.

Figure 6d,e demonstrate that two different tensile cycles in the strain range of 0–100% were tested by IHSS, respectively. Each strain range can be detected and quantitatively distinguished because of the good linear-resistance variation of the sensor during stretching. In addition, repeatable curves are observed in each strain range due to the high robustness and stability of the sensor.

### 2.4. IHSS Used for Human Motion Monitoring

**Figure 7:**
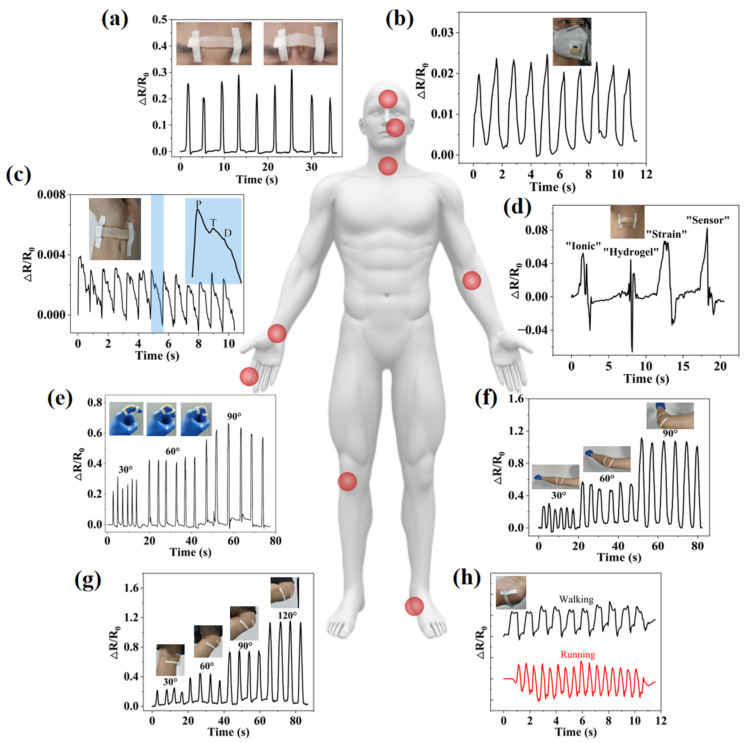
IHSS for real-time monitoring of human motions. (**a**) Monitoring frowning movement by installing the IHSS on the forehead. (**b**) Monitoring human breathing by integrating the IHSS on a mask. (**c**) Real-time radial artery pulse monitoring by attaching the IHSS to the wrist. The right enlarged is one complete radial artery pulse waveform containing “P”, “T”, and “D” peaks. (**d**) Sound and speech recognition by attaching the IHSS to the throat to detect its vocal-cord vibrations. (**e**) IHSS fixed on a finger that repeated bending/unbending at different angles (30°, 60°, and 90°), (**f**) elbow (30°, 60°, and 90°), and (**g**) knee (30°, 60°, 90°, and 120°). (**h**) IHSS fixed on the heel to detect the foot-movement states.

The human body is a multi-sensory system, and different parts have unique physiological signal characteristics. Based on good sensitivity and conformal capabilities, IHSS can monitor various forms of human physiological signals in a fast, real-time, non-invasive, and user-interactive manner for medical diagnosis and disease prevention (Figure 7). For example, IHSS can be attached to the facial muscles, such as on the brow, to recognize facial expressions. Repeatable ∆*R*/*R*_0_ signals are monitored during furrow-and-frown alternating movements, as shown in Figure 7a. In addition, breathing is a significant vital sign that helps assess health conditions, such as sleep quality and mood changes, which can be assessed by monitoring the airflow during breathing [29,30]. In Figure 7b, IHSS is placed on the vent of a commercial mask to detect the breathing flow. A complete breathing cycle consists of inspirations and exhalations, which produce resistance signals that rise and fall, respectively. The pulse is often used as one of the key vital signs to assess a person’s physical state, which can be measured from the radial artery at the wrist. Figure 7c shows a pulse signal of a tester that was recorded by the IHSS attached to the wrist. When a typical wrist-pulse waveform is extracted, it can be found to contain three characteristic peaks: “P” (percussive wave), “T” (tidal wave), and “D” (diastolic wave), which correspond to the states of systolic and diastolic blood pressure, ventricular pressure, and heart rate, respectively. In addition, IHSS also has the capability of speech recognition, which is based on the vibration of the throat during the speech process to obtain the *∆R/R*_0_ signal. For example, in Figure 7d, when the subject said “ionic”, the image showed characteristic peaks and valleys. According to the differences in the syllable, length, and weight, the words “ionic”, “hydrogel”, “strain”, and “sensor” show differentiable characteristic peaks and valleys.

IHSS can be well conformed to the skin deformation when movement occurs, causing the sensor geometry to stretch or contract and the resistance to change accordingly, even in the case of large deformations. Figure 7e shows the changes in the ∆*R*/*R*_0_ signals when an index finger is gradually repeatedly bent to 30°, 60°, and 90°. The ∆*R*/*R*_0_ signal increased with the increase of the finger-bending angle. Due to the good resilience of the IHSS, the ∆*R*/*R*_0_ signal immediately returned to its initial value when the finger was straightened again. Much larger strains induced by bending of the elbow (Figure 7f) and the knee (Figure 7g) were also tracked from variation in ∆*R*/*R*_0_. The relative resistance gradually increased as the bending angle increased from 0° to 90° and from 0° to 120°. While keeping the angle of the joints’ bending constant, the signals are stable, indicating that the measurement of joints’ movements is immediate, accurate, and long-lasting. In addition, due to the excellent strength and robustness of the hydrogel itself and the PDMS coating, IHSS can withstand and be fixed at the heel to detect the movement states of the feet. As shown in Figure 7h, the amplitude and frequency of the piezoelectric signals generated by walking and running are different and can be well distinguished.

## 3. Conclusions

In summary, we developed a simple and general strategy for realizing the strong adhesion between hydrogels and elastomers (PDMS) to avoid dehydration of the hydrogels. The adhesion is achieved by using porous structured thermoplastic polyurethane (TPU) at the hydrogel-elastomer interface to interlock the hydrogel and bond the elastomer simultaneously. A PVA-G-FeCl_3_ hydrogel was developed as a strain sensor with the encapsulation of PDMS. The effect of G and hygroscopic salt, FeCl_3_ on water retention, and other mechanical and electrical properties of the hydrogel was studied. The encapsulated IHSS exhibits an excellent water-retention ability, superior mechanical performance, highly linear sensitivity, and robust interface. At a FeCl_3_ molar mass of 0.4 M, the ionic hydrogel reached the highest conductivity (2.15 S/m) and GF (2.21), which enables highly linear strain sensitivity under stretching and a high potential for skin-sensor applications. Large strains, such as human joint-bending detection, and minor strains, such as human pulse, respiration, vocal cord vibration, and facial muscle expression, were monitored. The results reveal the practicability and effectiveness of combining hydrogel with elastomer by porous TPU to realize advanced ionic electronics with better robustness and longevity and for many valuable applications in human-motion monitoring and so on.

## 4. Materials and Methods

Materials: Poly(vinyl alcohol) (PVA) (average Mw 130,000, 99+% hydrolyzed; Sigma-Aldrich, Saint Louis, MO, USA), G (ACS, ≥99.5%; Aladdin, Shanghai, China), FeCl_3_·6H_2_O (Aladdin, Shanghai, China), NaCl (AR, 99.5%; Aladdin, Shanghai, China), PDMS-based elastomer (Sylgard184; Dow corning, Midland, Michigan, USA), and TPU (Elastollan 35A, BASF, Ludwigshafen, Germany). Unless otherwise specified, the water used in this experiment is deionized water (MΩ at 25 °C).

Fabrication of PVA10-G30-FeCl_3_ hydrogel solution: All the PVA-based hydrogels were prepared by one time of freezing and thawing. Firstly, 10 wt.% of PVA powder was dissolved in a mixture of deionized water and G (10 wt.%, 20 wt.% or 30 wt.%) under heating (100 °C) and magnetic stirring for 2 h. After that, 0.4 M FeCl_3_ particles were added and dissolved in the PVA-G solution.

Fabrication of porous TPU: First of all, TPU slurry was mixed with NaCl particles, uniformly, at a mass ratio of 1:8, and formed by a molding method. After heat treatment (100 °C, 2 h) and removal of NaCl (immersed in water for 12 h), the porous TPU layer was obtained.

Fabrication of PDMS elastomer layer: The curing agent and silicone base were mixed by hand for 1 min with the weight ratios of 1:10, 1:15, and 1:20. Then, the mixtures were poured into a mold and cured at 100 °C for 1 h in an oven.

Fabrication of the IHSS: The porous TPU layer was placed on the uncured PDMS (the ratio of crosslinker to monomer is 1:20), and they were heated to 100 °C together for an hour. During PDMS curing, the porous TPU can be embedded on its surface and bonded together. Then the un-crosslinked PVA10-G30-FeCl_3_ solution was spread on the TPU layer. Under the influence of gravity, the flowing hydrogel can soak into the micropores on the surface of the TPU layers, resulting in a large interfacial area. After frozen crosslinking (−20 °C, 12 h), the hydrogel can be mechanically interlocked at the surface of the porous TPU layers to achieve good adhesion.

SEM characterization: For the characterization of the micro- and nanostructures of the PVA-G hydrogel, all hydrogel samples (70 mm × 10 mm ×1.2 mm) were freeze-dried for 48 h by using a freeze drier. The freeze-dried hydrogels were then cut to expose the inside and sputtered with gold before carrying out the imaging, using SEM.

Mechanical tests: The mechanical properties of PDMS and hydrogels were tested by using an INSTRON 5943 universal testing machine. The length, width, and thickness of all stretched samples were 70 mm × 10 mm ×1.2 mm, and the stretching rate was 0.5 mm/s. The Young’s modulus (*E*) was calculated by dividing the applied stress (σ) by the applied strain (ε):

(2) $$E=\frac{\sigma }{\varepsilon }=\frac{F/S}{\Delta L/L_{0}}$$

where *F* is the tensile force, *S* is the cross-sectional area, Δ*L* is the variation of the length, and *L*_0_ is the initial length of the sample.

Conductivity measurement: The PVA10-G30-FeCl_3_ hydrogel samples with different ion concentrations (0 M, 0.1 M, 0.2 M, 0.3 M, and 0.4 M) were connected to an LCR meter (Keysight E4980AL, Solon, OH, USA) to measure the resistance at a frequency of 50 Hz, which was then used to calculate the resistivity of the hydrogel.

Electrical tests: A digital multimeter (Keithley DMM6500, Solon, OH, USA) was used to measure the electrical signal of the hydrogels. When the hydrogel sample (70 mm × 10 mm × 1.2 mm) was stretched, two electrodes were attached at both ends of it and connected to the multimeter. The change of resistance with time was obtained as the sample was stretched at the speed of 0.5 mm/s.

## Data Availability

The data presented in this study are available upon request from the corresponding author.
